# Preoperative chemotherapy combined with para-aortic lymph node dissection has clinical value in the treatment of gastric cancer with para-aortic lymph node metastases

**DOI:** 10.1186/s12893-022-01844-0

**Published:** 2022-11-20

**Authors:** Pengcheng Yu, Can Hu, Yi Wang, Zhehan Bao, Ruolan Zhang, Mengxuan Cao, Yanqiang Zhang, Xiangdong Cheng, Zhiyuan Xu

**Affiliations:** 1 The First Clinical Medical College of Zhejiang, Chinese Medical University, Hangzhou, 310053 China; 2 The Second Clinical Medical College of Zhejiang, Chinese Medical University, Hangzhou, 310053 China; 3 Wenzhou Medical University, Wenzhou, 325035 China; 4 The Cancer Hospital of the University of Chinese Academy of Sciences (Zhejiang Cancer Hospital), Institutes of Basic Medicine and Cancer (IBMC), Chinese Academy of Sciences, Banshan Road 1#, Hangzhou, 310022 China

**Keywords:** Gastric cancer, Para-aortic lymph node dissection, Para-aortic lymph node metastasis, Chemotherapy

## Abstract

**Background:**

Lymph node metastases often occur in advanced gastric cancer, with some patients presenting with metastases in the para-aortic lymph nodes. There are persistent Controversies about the benefit of para-aortic lymph node dissection (PAND). Our purpose is to probe whether PAND following preoperative chemotherapy had any clinical significance in individuals with PALNs in gastric cancer.

**Material and methods:**

To retrospectively analyze the clinical data of 86 gastric cancer patients (40 in the D2 + PAND group and 46 in the D2 group) who attended the abdominal surgery department of Zhejiang Cancer Hospital between September 1, 2008, and July 30, 2018.

**Results:**

In the D2 + PAND group (40 cases), the average number of lymph nodes cleared per case was 4.3 in group 16 (16a2, 16b1), and the postoperative pathology confirmed lymph node positivity in 16 cases, with a metastasis rate of 40%. The median overall survival times were 63 and 34 months for the patients in the D2 + PAND group and D2 group, respectively. The 3-year overall survival (OS) compared to the D2 group (D2 + PAND 69.1% vs. D2 50%, P = 0.012) and a statistically significant difference in 3-year disease-free survival (DFS) (D2 + PAND 69.6% vs. D2 38.3%, P = 0.007). Lymph node dissection extent and recurrence of para-aortic lymph nodes were independent prognostic variables for the patients. The recurrence rate was reduced in the D2 + PAND group compared to the D2 group (D2 + PAND 7.5% vs. D2 26.1%, p = 0.023).

**Conclusions:**

For patients with gastric cancer whose imaging suggests metastasis in the para-aortic lymph nodes, preoperative chemotherapy combined with PAND is an effective and safe treatment that may benefit patient survival.

## Introduction

According to the World Health Organization’s 2020 data report, China has approximately 480,000 new instances of gastric cancer (GC) and ranks third in the world in terms of new malignant tumors, with 370,000 deaths from malignant tumors, ranking third in the world [1]. Advanced gastric cancer is the most common type of stomach cancer in China, accounting for approximately 80% of the cases.

According to some studies, the metastatic rate of advanced gastric cancer para-aortic lymph nodes (PALNs) can range from 18 to 40% [2–5]. The efficacy of para-aortic lymph node dissection (PAND) for PALN metastases has been controversial. The 3rd version of the Japanese Guidelines for the Treatment of Gastric Cancer defines PALN as M1 based on the Japanese JCOG9501 trial, which denied the therapeutic efficacy of prophylactic D2 + PAND [6, 7]. Based on the outcomes of the JCOG0001 and JCOG0405 investigations, Japanese researchers now consider that preoperative chemotherapy followed by D2 + PAND in patients with circumscribed para-aortic lymph node metastases from gastric cancer can benefit patients with a 5-year overall survival (OS) of 53% [8, 9]. The follow-up endpoint of a Chinese phase II clinical study of D2 lymph node dissection alone after chemotherapy for gastric cancer with PALN metastases was not fulfilled, but the median survival time has now reached 29.8 months [10]. However, other studies have concluded that the use of neoadjuvant chemotherapy before minimally invasive radical gastrectomy with D2 lymph node dissection does not increase postoperative complications [11]. Patients with ypN0 have a good prognosis because the lymph nodes are indeed negative before neoadjuvant chemotherapy or because the pathology achieves a complete response after treatment [12]. Neoadjuvant chemotherapy appears to be associated with a higher rate of postoperative complications compared to surgery alone [13]. Currently, the clinical significance of preventative D2 + PAND surgery has been ruled out, but the clinical significance of curative D2 + PAND surgery is unknown.

We retrospectively analyzed the clinical and pathological data of patients with gastric cancer who were found to have metastatic lymph nodes in the parietal abdominal aorta 16a2 and 16b1 regions at the initial CT diagnosis and who underwent radical gastric cancer surgery after preoperative chemotherapy. The goal of this research was to probe whether parietal aortic lymph node dissection following preoperative chemotherapy had any clinical significance in individuals with metastatic parietal aortic lymph nodes in gastric cancer.

## Patients and methods

### Selection criteria and patients

We retrospectively analyzed the clinical and pathological data of consecutive patients with histologically confirmed GC/Esophagogastric junction cancer (EGJC) and who received surgical treatment at the Department of Abdominal Oncology Surgery at Zhejiang Cancer Hospital between September 1, 2008, and July 30, 2018. The inclusion criteria were as follows: (1) The pathological examination from biopsy confirmed gastric adenocarcinoma. (2) A preoperative CT revealed enlarged lymph nodes in the 16a2 and 16b1 areas adjacent to the abdominal aorta. (3) The patient had normal function of major organs. (4) The patient received at least 3 cycles or more of first-line chemotherapy before surgery (chemotherapy regimen was not limited). (5) Radical gastric cancer surgery was performed (R0 excision). The criteria for exclusion were as follows: (1) previous gastric surgery; (2) associated with other malignant tumors; (3) other distant metastases; (4) received radiotherapy; (5) unable to tolerate chemotherapy and surgery; and (6) incomplete data. Finally, 86 patients were included in this study.

According to the extent of lymph node dissection after surgery, the patients were classified into 40 patients in the D2 + PAND group (observation group) and 46 patients in the D2 group (control group). Before surgery, all patients had a gastroscopy to confirm that they had stomach cancer, and CT and B-mode ultrasound were used to rule out distant metastases such as metastases to the supraclavicular lymph nodes, liver, and peritoneum. There were 61 men and 25 women among the patients, with a median age of 59 (28–74) years. The patients’ clinical data, such as tumor location, surgical method, lymph node dissection extent, anastomotic method, and postoperative complications, were also collected.

This study was approved by the ethics committee of the Cancer Hospital of the University of Chinese Academy of Sciences (Zhejiang Cancer Hospital) (No. IRB-2020-300). Informed consent from the patients was waived by the ethics committee of the Cancer Hospital of the University of Chinese Academy of Sciences (Zhejiang Cancer Hospital) (No. IRB-2020-300) because of the retrospective nature of this study, and conformed to the tenets of the Declaration of Helsinki (as revised in 2013).

### Treatment method

All patients received 3 or more cycles of preoperative chemotherapy, including SOX (oxaliplatin + S-1) in 44, DOS (docetaxel + oxaliplatin + S-1) in 4, PS (paclitaxel + S-1) in 14, EOX (epirubicin + oxaliplatin + capecitabine) in 2, ECX (epirubicin + cisplatin + capecitabine) in 9, XELOX (oxaliplatin + capecitabine) in 9, FOLFOX (oxaliplatin + fluorouracil + calcium folinate) in 1, and DCF (docetaxel + cisplatin + fluorouracil) in 3. After evaluation of the efficacy of the preoperative chemotherapy, radical surgery for D2 gastric cancer was considered when the lymph node size after chemotherapy was suggested by CT and when the metastasis next to the abdominal aorta disappeared or shrunk to < 1.0 cm. When the metastatic lymph nodes adjacent to the abdominal aorta were > 1.0 cm, radical surgery for D2 + PAND gastric cancer was considered.

**Fig. 1:**
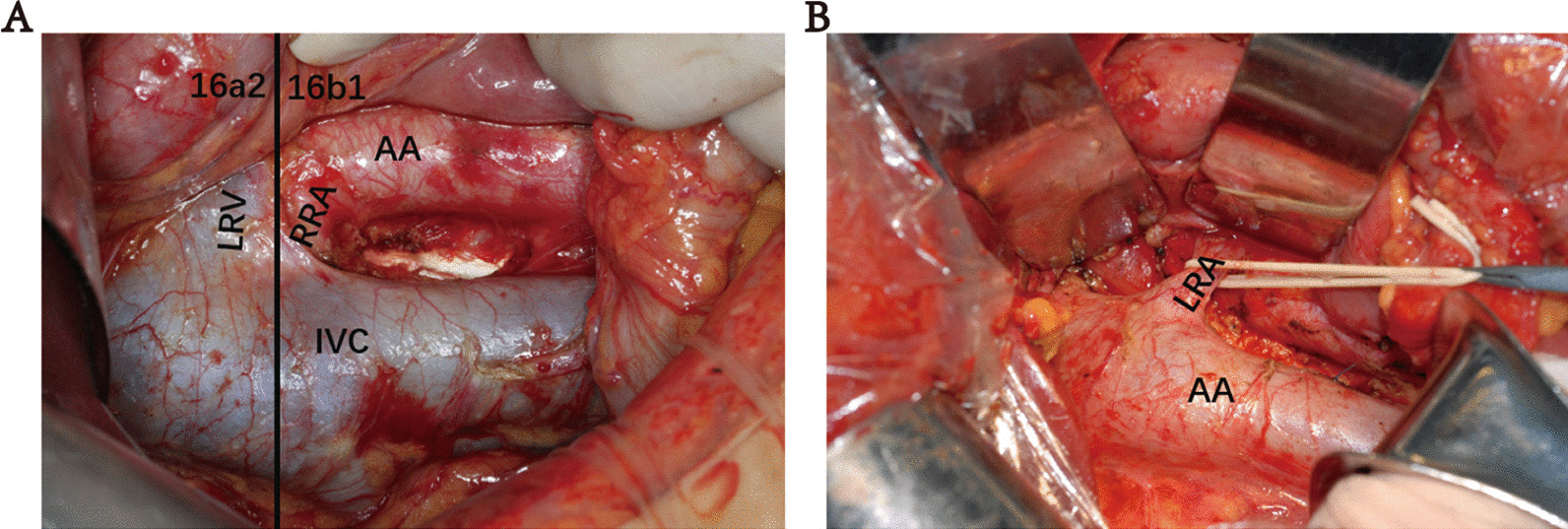
Post-abdominal para-aortic lymph node dissection. **A** Right 16a2, 16b1; **B** left 16a2, 16b1. *LRV* left renal vein, *RRA* right renal artery, *AA* abdominal aorta, *IVC* inferior vena cava, *LRA* left renal artery

Intraoperative lymph node dissection of the para-aortic lymph nodes: the transverse colonic hepatic flexure was freed inward and downward, Kochers’ maneuver was made, the left hemicocele was freed and turned up to the right, the posterior part of the pancreas was freed up to the left side of the abdominal aorta, and the 16a2, 16b1 lymph nodes were cleared from left to right, starting from the superior border of the abdominal aortic trunk up to the superior border of the inferior mesenteric artery. The fine lymphatic vessels were meticulously ligated or sutured (Fig. 1A, B).

### Assessment and follow-up

Following surgery, all patients were followed up every 3 months for the first year, every 6 months for the next 1 to 3 years, and every 12 months after 3 years. Outpatient and telephone follow-ups were used, and each outpatient review included an assessment. For the evaluation: a thorough medical history, physical examination, serum tumor markers, and radiographic exams (enhanced CT or PET-CT) were obtained. Assessment of PALNs for recurrence (enlarged lymph nodes ≥ 1 cm in diameter present in the para-aortic abdomen) by enhanced CT or pet-ct findings. The patients were followed for 3 to 142 months, with a median follow-up period of 34 (20, 51.25) months. The final follow-up date was November 10, 2021.

### Statistical methods

GraphPad Prism 8.0 software was used for statistical analysis. The rank sum test was used to compare measurement data. The chi-square test was used to compare count data, which were expressed as the number of cases (n) and the rate (%). The Kaplan–Meier method was used to plot survival curves, and the log-rank approach was employed to test significance. For univariate and multifactorial prognostic analyses, Cox models were applied. The differences were judged to be statistically significant at P < 0.05.

## Results

### Clinicopathological characteristics and clinical results

This study included 61 (70.9%) males and 25 (29.1%) females; the patient’s median age was 59 (28–74) years. Additionally, 40 (46.5%) patients had enlarged lymph nodes in zone 16a2; 46 (53.5%) patients did not have enlarged lymph nodes in zone 16a2. Also, 60 (69.8%) patients had enlarged lymph nodes in zone 16b1; 26 (30.2%) patients did not have enlarged lymph nodes in zone 16b1. There were 67 (77.9%) patients with two-drug combination, and 19 (22.1%) patients with three-drug combination. Thirteen (15.1%) patients had a tumor located in the proximal stomach; 26 (30.2%) patients had tumors in the gastric body; 43 (50%) patients had tumors in the distal stomach; and 4 (4.7%) patients had tumors in the whole stomach. There were 10 Borrmann type I patients (11.6%), 54 Borrmann type II patients (62.8%), 13 Borrmann type III patients (15.1%), and 9 Borrmann type IV patients (10.5%). There were 54 (62.8%) patients with hypofractionation, 32 (37.2%) patients with intermediate differentiation, 39 (45.3%) patients with neurological invasion and 47 (54.7%) patients without neurological invasion. The median OS was 35 (4–142) months; and 49 (56.98%) patients died during the follow-up period.

### Comparison of clinicopathological characteristics between the patients in the D2 + PAND group and D2 group

Between September 1, 2008, and July 30, 2018, 86 patients were eligible for enrollment. In the D2 + PAND group, there were 40 patients, while in the D2 group, there were 46 patients. Sex, age, enlarged lymph nodes in the first 16a2 and 16b1 areas, chemotherapy regimen, tumor location, Borrmann type, grade of differentiation, and level of neurological invasion were not significantly different between the two groups (Table 1).

**Table 1:** Comparison of clinical characteristics of patients in D2 + PAND group and D2 group

Variables	Scope of lymph node dissection		χ^2^	p-value
	D2 + PAND (n = 40)	D2 (n = 46)		
Sex			0.427	0.514
Male	27 (67.5%)	34 (73.9%)		
Female	13 (32.5%)	12 (26.1%)		
Age (year)			0.054	0.817
< 60	21 (52.5%)	23 (50%)		
≥ 60	19 (47.5%)	23 (50%)		
16a2 area			3.629	0.057
Negative	17 (42.5%)	29 (63.0%)		
Positive	23 (57.5%)	17 (37.0%)		
16b1 area			2.120	0.145
Negative	9 (22.5%)	17 (37.0%)		
Positive	31 (77.5%)	29 (63.0%)		
Chemotherapy regimens			0.367	0.545
Dual drug combination	30 (75%)	37 (80.4%)		
Triple drug combination	10 (25%)	9 (19.6%)		
Tumor location			4.737	0.192
Proximal stomach	3 (7.5%)	10 (21.7%)		
Middle stomach	14 (35%)	12 (21.7%)		
Distal stomach	20 (50%)	23 (54.3%)		
Total stomach	3 (7.5%)	1 (2.2%)		
Borrmann type			0.863	0.834
Borrmann I	10 (11.6%)	6 (13%)		
Borrmann II	54 (62.8%)	28 (28%)		
Borrmann III	13 (15.1%)	8 (17.4%)		
Borrmann IV	9 (10.5%)	4 (8.7%)		
Grade of differentiation			0.156	0.693
Poor	26 (65%)	28 (60.9%)		
Moderate	14 (35%)	18 (39.1%)		
Nerve invasion			0.140	0.709
Negative	21 (52.5%)	26 (56.5%)		
Positive	19 (47.5%)	20 (43.5%)		

### Comparison of the surgical modalities and complications between the D2 + PAND group and the D2 group

In terms of the surgical resection range, reconstruction, intraoperative hemorrhage, and postoperative hospital length of stay, there was no statistically significant difference between the D2 + PAND group and the D2 group. The D2 + PAND group had a median time to surgery of 198 (189, 205.5) min, while the D2 group had a median time to surgery of 167 (156, 178) min. There was a statistically significant difference in the median time to surgery between the two groups (Z = 7.138, P = 0.001) (Table 2).

**Table 2:** D2 + PAND group compared with D2 group for surgery

Variables	Scope of lymph node dissection		p-value	
	D2 + PAND (n = 40)	D2 (n = 46)		
Excision extension			0.574	χ^2^ = 0.317
Distal gastrectomy	15 (37.5%)	20 (43.5%)		
Total gastrectomy	25 (62.5%)	26 (56.5%)		
Reconstruction method			0.142	χ^2^ = 3.898
Roux-en-Y	19 (47.5%)	26 (56.5%)		
Billronth I	4 (10%)	9 (19.6%)		
Billronth II	17 (42.5%)	11 (23.9%)		
Intraoperative bleeding (ml)	200 (100, 275)	175 (100, 200)	0.270	Z = 1.104
Surgery time (min)	198 (189, 205.5)	167 (156, 178)	< 0.001*	Z = 7.138
Hospitalization time (day)	12 (9.25, 13)	11 (9, 13.25)	0.827	Z = 0.218

*Statistically significant (p < 0.05)

The D2 + PAND group had one case of intestinal blockage, two cases of celiac leakage, one case of abdominal infection, and one case of anastomotic leakage. In the D2 group, there was one instance of duodenal stump leakage, one case of celiac leakage, one case of abdominal infection, two cases of anastomotic leakage, and two cases of pulmonary infection; the frequency of postoperative complications was not statistically significant (χ^2^ = 0.132, P = 0.717). There were 3 patients who had postoperative recurrence of abdominal para-aortic lymph nodes in the D2 + PAND group, 12 patients in the D2 group, and this was a statistically significant difference (χ^2^ = 5.133, P = 0.023) (Table 3).

**Table 3:** Comparison of postoperative complications and recurrence of para-aortic lymph nodes in D2 + PAND group and D2 group

Variables	Scope of lymph node dissection		χ^2^	p-value
	D2 + PAND (n = 40)	D2 (n = 46)		
Complication			0.132	0.717
Negative	35 (87.5%)	39 (84.8%)		
Positive	5 (12.5%)	7 (15.2%)		
Lymph node recurrence			5.133	0.023*
Negative	37 (92.5%)	34 (73.9%)		
Positive	3 (7.5%)	12 (26.1%)		

*Statistically significant (p < 0.05)

### Lymph node metastasis in 16 groups

In the D2 + PAND group (40 patients), 172 lymph nodes were eliminated in 16 groups (16a2, 16b1), with an average of 4.3 lymph nodes cleared per case. Postoperative pathology revealed 63 positive lymph nodes. The lymph node-positive group had 16 instances, while the lymph node-negative group had 24 cases. There was a 40% metastatic rate.

### *Prognostic analysis of the D2* + *PAND group versus D2 group*

**Fig. 2:**
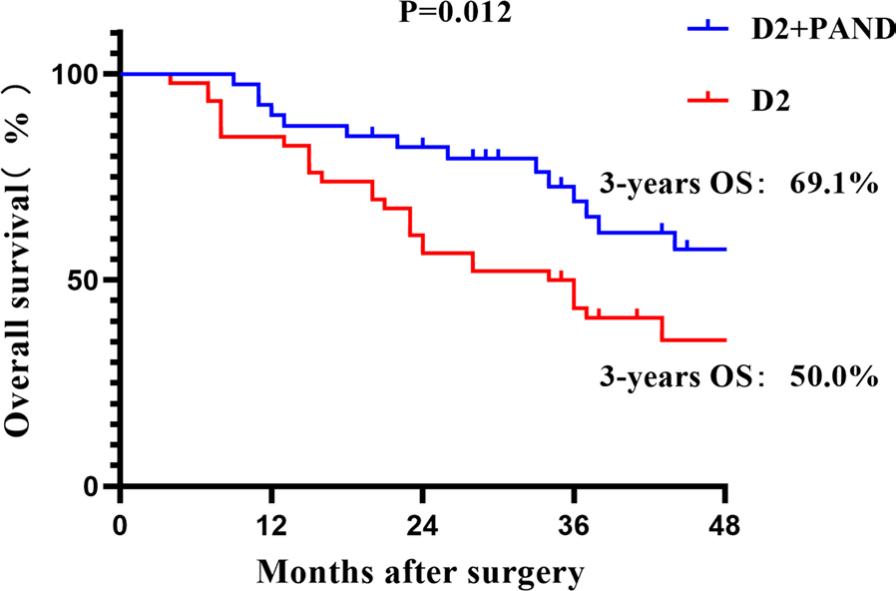
Overall survival between D2 + PAND and D2 groups after preoperative chemotherapy

**Fig. 3:**
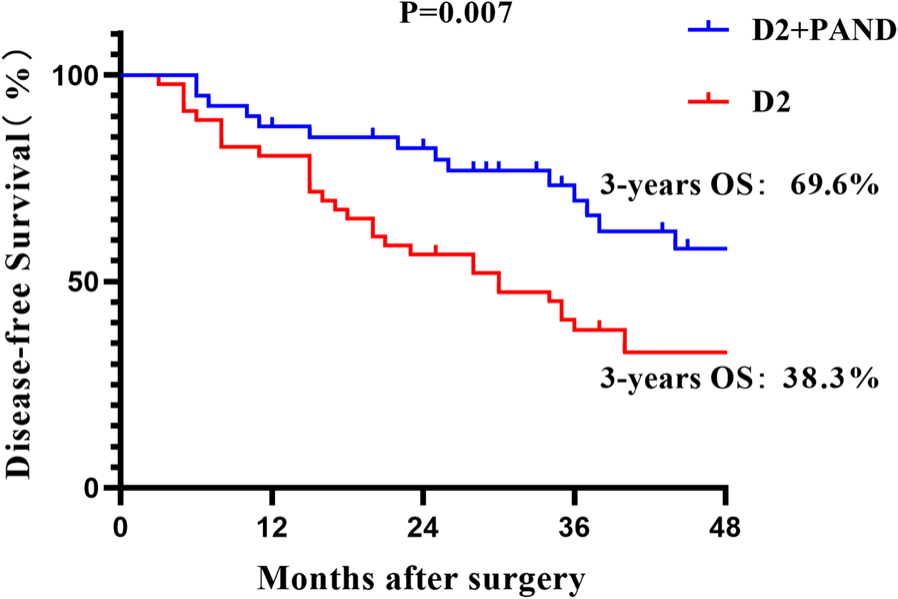
Disease-free survival between D2 + PAND and D2 groups after preoperative chemotherapy

The patients in the D2 + PAND group had a statistically significant difference in their 3-year OS compared to the D2 group (D2 + PAND 69.1% vs. D2 50%, P = 0.012) (Fig. 2) and a statistically significant difference in 3-year disease-free survival (DFS) (D2 + PAND 69.6% vs. D2 38.3%, P = 0.007) (Fig. 3). The degree of lymph node dissection, nerve invasion, and recurrence of para-aortic lymph nodes in the abdomen were all linked to the patient overall survival in the univariate Cox analysis. In the multifactorial Cox analysis, nerve invasion (HR = 1.982 95% CI 1.012 to 3.882 P = 0.046), lymph node dissection extent (HR = 0.422 95% CI 0.212 to 0.840 P = 0.014), and recurrence of para-aortic lymph nodes (HR = 2.488 95% CI 1.128 to 5.489 P = 0.024) were all found to be Independent factors affecting overall patient survival in this study (Table 4).

**Table 4:** Prognostic factors in univariate and multivariate analyses

Factors	Univariate analysis		Multivariate analysis	
	HR (95% CI)	P-value	HR (95% CI)	P-value
Sex				
Male	1			
Female	1.479 (0.802–2.727)	0.211		
Age (year)				
< 60	1			
≥ 60	1.016 (0.580–1.781)	0.954		
16a2 area				
Negative	1			
Positive	1.120 (0.639–1.964)	0.691		
16b1 area				
Negative	1			
Positive	0.913 (0.502–1.660)	0.765		
Chemotherapy regimens				
Dual drug combination	1			
Triple drug combination	1.069 (0.557–2.052)	0.840		
Tumor location				
Proximal stomach	1			
Middle stomach	0.773 (0.320–1.869)	0.568		
Distal stomach	0.997 (0.450–2.205)	0.993		
Total stomach	0.971 (0.206–4.584)	0.970		
Borrmann type				
Borrmann I	1			
Borrmann II	0.764 (0.293–1.994)	0.582		
Borrmann III	1.918 (0.674–5.460)	0.223		
Borrmann IV	0.882 (0.255–3.055)	0.843		
Grade of differentiation				
Poor	1			
Moderate	0.586 (0.318–1.079)	0.086		
Nerve invasion				
Negative	1		1	
Positive	2.248 (1.269–3.982)	0.005*	2.493 (1.400–4.437)	0.002*
Excision extension				
Distal gastrectomy	1			
Total gastrectomy	1.205 (0.678–2.141)	0.526		
Lymph node dissection extent				
D2	1		1	
D2 + PAND	0.472 (0.259–0.859)	0.014*	0.514 (0.269–0.981)	0.044*
Reconstruction method				
Roux-en-Y	1			
Billronth I	1.184 (0.571–2.458)	0.650		
Billronth II	0.766 (0.393–1.493)	0.434		
Surgery time				
< 180 min	1			
≥ 180 min	0.671 (0.382–1.178)	0.164		
Intraoperative bleeding				
< 200 ml	1			
≥ 200 ml	0.829 (0.470–1.461)	0.516		
Hospitalization time				
< 12 days	1			
≥ 12 days	1.162 (0.662–2.038)	0.601		
Complication				
Negative	1			
Positive	1.235 (0.575–2.654)	0.588		
Lymph node recurrence				
Negative	1		1	
Positive	2.740 (1.421–5.285)	0.003*	2.146 (1.066–4.319)	0.032*

*Statistically significant (p < 0.05)

## Discussion

In the JCOG9501 trial, the Japanese Society of Clinical Oncology (SCSG/JCOG) found no survival benefit from prophylactic D2 + PAND compared to D2 lymph node dissection alone in patients with advanced gastric cancer [7]. In this study, 523 patients with advanced gastric cancer were recruited to examine the prognostic impact of D2 + PAND surgery vs. D2 surgery alone. According to the findings here, there was no significant difference in 5-year recurrence-free survival or 5-year overall survival. The D2 + PAND group, on the other hand, had a higher rate of postoperative complications. As a result, the use of preventive D2 + PAND in the treatment of advanced gastric cancer has been denied. After excluding the patients with a leathery stomach or who had more than 15 positive lymph nodes, Tokunaga et al. [14] found that the 5-year OS after D2 + PAND clearance reached 28.6% in patients with positive para-abdominal aortic lymph nodes. The 5-year OS after D2 + PAND was just 17% in a study by the Italian Gastric Cancer Research Group (GIRCG) [15]. In all of the investigations, D2 + PAND did not improve the survival in individuals with gastric cancer and who had metastases to the para-aortic lymph nodes.

Chemotherapy followed by D2 + PAND improves the prognosis in individuals with focal para-aortic lymph node metastases from gastric cancer. JCOG conducted three phase II clinical trials (JCOG0001, JCOG0405, and JCOG1002). to determine whether preoperative chemotherapy combined with D2 + PAND is effective and safe in patients with advanced gastric cancer. In the JCOG0001 experiment [9], the patients were given two or three cycles of irinotecan and cisplatin, followed by D2 + PAND gastrectomy. The 3-year survival rate was 27%. Following that, 53 gastric cancer patients were enrolled in the JCOG0405 trial [8], which had similar enrollment criteria to the JCOG0001 trial. Preoperative chemotherapy with S-1 and cisplatin was given to all of the patients. By using the same examination for all patients, a gastrectomy with D2 + PAND was performed, and the R0 resection rate was 82%. Their findings revealed a 64.7% RR, a 58.8% 3-year survival rate, and a low frequency of major adverse events with no treatment-related fatalities. In comparison to the JCOG0405 study, preoperative DCS (doxorubicin, cisplatin, and S-1) in the JCOG1002 study failed to produce sufficient efficiency in patients with extensive lymph node metastases [16]. Based on the results of these phase II trials, S-1 in combination with cisplatin was considered more effective than irinotecan in combination with cisplatin. The 5th edition of the Japanese guidelines for gastric cancer also suggests a combination of preoperative chemotherapy combined with D2 + PAND surgery for patients with lymph node metastasis in the NO. 16a2/b1 group alone [17].

Preoperative chemotherapy (capecitabine and oxaliplatin) followed by a combined D2 gastrectomy had adequate R0 resection rates, according to a study by Chinese researchers [10]. Another real-world study found that D2 gastrectomy alone is safe and effective in patients with gastric cancer who have metastatic abdominal para-aortic lymph nodes and respond well to preoperative chemotherapy [18]. Nonetheless, for patients with gastric cancer with para-aortic lymph node metastases, no relevant controlled studies have directly revealed the advantages and disadvantages of preoperative chemotherapy followed by D2 + PAND surgery over D2-only surgery. As a result, we undertook this study to explore whether preoperative chemotherapy combined with paraaortic lymph node dissection had any therapeutic value in the treatment of patients with gastric cancer and paraaortic lymph node metastases.

In this study, there were no statistically significant differences between the D2 + PAND group and the D2 group in terms of sex, age, preoperative chemotherapy regimen, tumor location, tumor differentiation, or other characteristics, and the two groups were comparable. In comparison to conventional D2 radical gastric cancer surgery, D2 + PAND radical gastric cancer surgery was more difficult and required more operative time, but there were no significant differences in the intraoperative bleeding, postoperative complication rate, or postoperative length of hospital stay between the groups. Seckin et al. [19] found that D2 + PAND surgery, when compared to D2 surgery, did not enhance the length of postoperative hospital stay or the rate of postoperative complications. Other research has found that a routine D2 + PAND surgery can be conducted safely and with no increase in the postoperative mortality [20]. We believe that D2 + PAND surgery is safe and practical and that it can be conducted in specialist centers and has a low surgical risk. However, D2 + PAND radical gastric cancer surgery takes longer to complete than D2 radical gastric cancer surgery. The intricate retroperitoneal anatomy that is involved with the removal of the para-aortic lymph nodes, the restricted intraoperative view, and the potential for blood vessel damage may all play a role in its difficulty. D2 + PAND should only be performed in a cancer center with skilled surgeons, as there are some dangers associated with the formation of complications, such as celiac fistula, in some rare cases.

Further prognostic analysis revealed that combined D2 + PAND radical surgery for gastric cancer after preoperative chemotherapy improved overall survival and disease-free survival compared to D2 radical surgery for gastric cancer. Also, the extent of lymph node dissection was an independent factor affecting overall survival. The overall survival rates of patients with gastric cancer with para-aortic lymph node metastasis after neoadjuvant chemotherapy combined with surgery were 80% and 48% at 1 and 3 years, respectively, and the disease-free survival rates were 72% and 38% at 1 and 3 years, according to the findings of a domestic study. This study found that neoadjuvant chemotherapy followed by surgery can dramatically improve these patients’ prognoses. Based on these findings, we infer that preoperative chemotherapy followed by D2 + PAND surgery may result in a survival benefit for patients with gastric cancer and metastatic abdominal para-aortic lymph nodes. The overall survival rates of patients with gastric cancer with para-aortic lymph node metastasis after neoadjuvant chemotherapy combined with surgery were 80% and 48% at 1 and 3 years, respectively, and the disease-free survival rates were 72% and 38% at 1 and 3 years, respectively, according to the findings of a Chinese study. This study found that neoadjuvant chemotherapy followed by surgery can dramatically improve these patients’ prognoses [21]. Based on the results of this study, we also infer that preoperative chemotherapy followed by D2 + PAND surgery may result in a survival benefit for patients with gastric cancer and metastatic abdominal para-aortic lymph nodes.

In our research, individuals with gastric cancer who were evaluated or paraaortic lymph node metastases based on CT at the initial visit had a postoperative pathologically confirmed metastatic rate of 40%. Furthermore, we discovered that the non-PAND group had a greater rate of PALN recurrence than the PAND group, and multifactorial analysis revealed that para-aortic lymph node recurrence was an independent risk factor for prognosis. Some studies have indicated a considerable reduction in the rate of recurrence in the retroperitoneal area in D2 + PAND patients [22]. They considered that D2 + PAND surgery for metastatic lymph nodes in the para-aortic area would be helpful. According to the abovementioned findings, eliminating the para-aortic lymph nodes in the abdomen may minimize the risk of retroperitoneal recurrence. Furthermore, paraaortic lymphatic recurrence in the abdominal aorta was found to be an independent prognostic factor in our study, which may also be the reason for the better OS in the D2 + PAND group than in the D2 group in our research.

As this study is a retrospective analysis, there are certain shortcomings: (1) the sample size of this study is small; (2) this study is a single-centre retrospective study and further prospective, multi-centre clinical studies are needed to confirm it. More prospective studies are needed to evaluate the optimal indication for D2 + PAND following preoperative chemotherapy in patients with gastric cancer as well as para-aortic lymph node metastases.

## Conclusion

In conclusion, we believe that the rate of PALN metastasis is higher in gastric cancer patients who have first-visit imaging that suggests paraaortic lymph node metastasis. Although D2 + PAND is a difficult procedure, preoperative chemotherapy combined with para-aortic lymph node dissection can improve overall survival, disease-free survival. And reduce the risk of retroperitoneal lymph node recurrence in patients with gastric cancer and para-aortic lymph node enlargement, and the procedure is safe and feasible.

## Data Availability

All data and materials are fully available without restriction. All data generated or analysed during this study are included in this published article.

## Abbreviations

PALNs: Para-aortic lymph nodes
PAND: Para-aortic lymph node dissection
GC: Gastric cancer
OS: Overall survival
DFS: Disease-free survival
EGJC: Esophagogastric junction cancer
SOX: Oxaliplatin + S-1
DOS: Docetaxel + oxaliplatin + S-1
PS: Paclitaxel + S-1
EOX: Epirubicin + oxaliplatin + capecitabine
ECX: Epirubicin + cisplatin + capecitabine
XELOX: Oxaliplatin + capecitabine
FOLFOX: Oxaliplatin + fluorouracil + calcium folinate
DCF: Docetaxel + cisplatin + fluorouracil
JCOG: Japanese Society of Clinical Oncology
GIRCG: Italian Gastric Cancer Research Group
